# Comparative Evaluation of Fracture Resistance of Teeth With Internal Resorption Cavities Repaired With Three Different Materials

**DOI:** 10.7759/cureus.115267

**Published:** 2026-08-26

**Authors:** Celin Gayose M, Fathima Mariyam, Gautham C Baiju, Sabir Muliyar, Mohammed Ashik P, Vishak Chand

**Affiliations:** 1 Conservative Dentistry and Endodontics, Malabar Dental College and Research Centre, Malappuram, IND

**Keywords:** fracture resistance, internal root resorption, mineral trioxide aggregate, root reinforcement, short fiber-reinforced composite

## Abstract

Introduction

Internal root resorption is a relatively uncommon pathological condition characterized by progressive loss of intraradicular dentin due to clastic cell activity. If left untreated, continued resorption weakens the remaining tooth structure, predisposing the tooth to perforation and fracture. Following endodontic management, repair of the resorptive defect using a material that provides both an effective seal and reinforcement of the weakened root is essential. Although mineral trioxide aggregate (MTA) is widely regarded as the material of choice because of its excellent biocompatibility and bioactivity, short fiber-reinforced composite (SFRC) has recently emerged as a promising alternative owing to its superior mechanical properties and crack-arresting ability.

Aim

To evaluate and compare the fracture resistance of simulated internal resorption defects repaired with SFRC, MTA, and gutta-percha (GP) (control).

Materials and methods

Thirty extracted human single-rooted teeth were instrumented, and standardized internal resorption defects were prepared. The specimens were randomly divided into three groups (n = 10): Group I, GP (control); Group II, МТА; and Group III, SFRC. Following repair of the defects, specimens were subjected to compressive loading in a universal testing machine until fracture. Fracture resistance values were recorded in Newtons and analyzed using one-way ANOVA and Tukey's HSD post hoc test (α = 0.05).

Results

The SFRC group exhibited the highest mean fracture resistance (1,476.29 ± 100.49 N), followed by the MTA group (1,021.78 ± 65.67 N), while the GP group demonstrated the lowest fracture resistance (829.78 ± 72.74 N). One-way ANOVA revealed a highly significant difference among the groups (F = 167.84, p < 0.001). Pairwise comparisons showed statistically significant differences between all groups.

Conclusion

Within the limitations of this in vitro study, repair of internal resorption defects using SFRC significantly improved fracture resistance compared with MTA and GP. SFRC may therefore represent a promising material for reinforcing teeth weakened by internal resorption.

## Introduction

Internal root resorption is an uncommon but potentially destructive pathological process characterized by progressive loss of intraradicular dentin caused by the activity of odontoclast-like cells [1,2]. The condition is generally associated with chronic pulpal inflammation following trauma, caries, restorative procedures, or other forms of pulpal injury [3,4]. Because the process is often asymptomatic during its early stages, diagnosis is frequently incidental on radiographic examination [5]. If left untreated, continued resorption may result in extensive dentinal destruction, root perforation, and eventual tooth fracture [1].

Successful management of internal resorption primarily depends on early diagnosis, elimination of inflamed pulp tissue through root canal treatment, and complete sealing of the resorptive defect [6,7]. However, the irregular morphology of internal resorption cavities poses a significant challenge for complete debridement and optimal adaptation of restorative materials [1,7]. Furthermore, the substantial loss of dentin compromises the structural integrity of the tooth, making reinforcement of the weakened root an important objective in treatment [8].

Various in vitro models have been developed to evaluate obturation materials in artificial internal resorption defects [9]. Mineral trioxide aggregate (MTA) has long been considered the gold standard for the repair of resorptive defects because of its excellent sealing ability, biocompatibility, bioactivity, and capacity to induce hard tissue formation [10-12]. Despite these advantages, MTA possesses relatively low flexural strength and brittle mechanical behavior, which may limit its ability to reinforce structurally compromised teeth [12,13]. Short fiber-reinforced composite (SFRC) has recently gained considerable attention as a restorative material because of its superior fracture toughness, high flexural strength, and crack-stopping mechanism provided by randomly oriented E-glass fibers within a resin matrix [14-16]. These fibers effectively dissipate functional stresses and inhibit crack propagation, thereby improving the fracture resistance of weakened teeth [15,17]. Owing to these favorable mechanical properties, SFRC has been proposed as a potential material for reinforcing teeth affected by extensive structural defects such as internal resorption [16,18].

Although previous studies have investigated the biological performance of MTA and the reinforcing ability of SFRC separately, evidence comparing these materials specifically for the repair of internal resorption defects remains limited [12,19,20]. Therefore, the present in vitro study was undertaken to evaluate and compare the fracture resistance of teeth with simulated internal resorption defects repaired using short SFRC, MTA, and gutta-percha (GP). The null hypothesis was that there would be no statistically significant difference in the fracture resistance of teeth with simulated internal resorption defects repaired using short SFRC, MTA, or GP.

## Materials and methods

Sample selection

Thirty freshly extracted human single-rooted permanent mandibular premolars with fully developed apices and similar root dimensions were selected. Teeth with root caries, fractures, cracks, previous endodontic treatment, internal or external resorption, calcifications, or developmental anomalies were excluded. Soft tissue remnants and calculus were removed using an ultrasonic scaler, and the teeth were stored in 0.1% thymol solution until use.

Sample size and grouping

Thirty specimens were randomly allocated into three experimental groups (n = 10 per group) based on the material used to repair the simulated internal resorption defects. In Group I, the defects were repaired with GP. In Group II, the defects were repaired using MTA, while in Group III, SFRC was used as the repair material.

Root canal preparation

Standardized endodontic access cavities were prepared using round diamond burs under copious water irrigation. Working length was established by inserting a #10 K-file until visible at the apical foramen and then subtracting 1 mm. Root canals were instrumented using EdgeFile X7 (EdgeEndo, Johnson City, TN) rotary nickel-titanium file system up to 25/0.06 taper. During instrumentation, canals were irrigated with 5.25% sodium hypochlorite (HYPOSOL; Prevest DenPro Pvt. Ltd., Jammu, India). Following preparation, 17% EDTA (Prevest DenPro Limited, India) was used for one minute to remove the smear layer, followed by a final rinse with distilled water. Canals were dried using sterile paper points.

Preparation of simulated internal resorption defects

**Figure 1:**
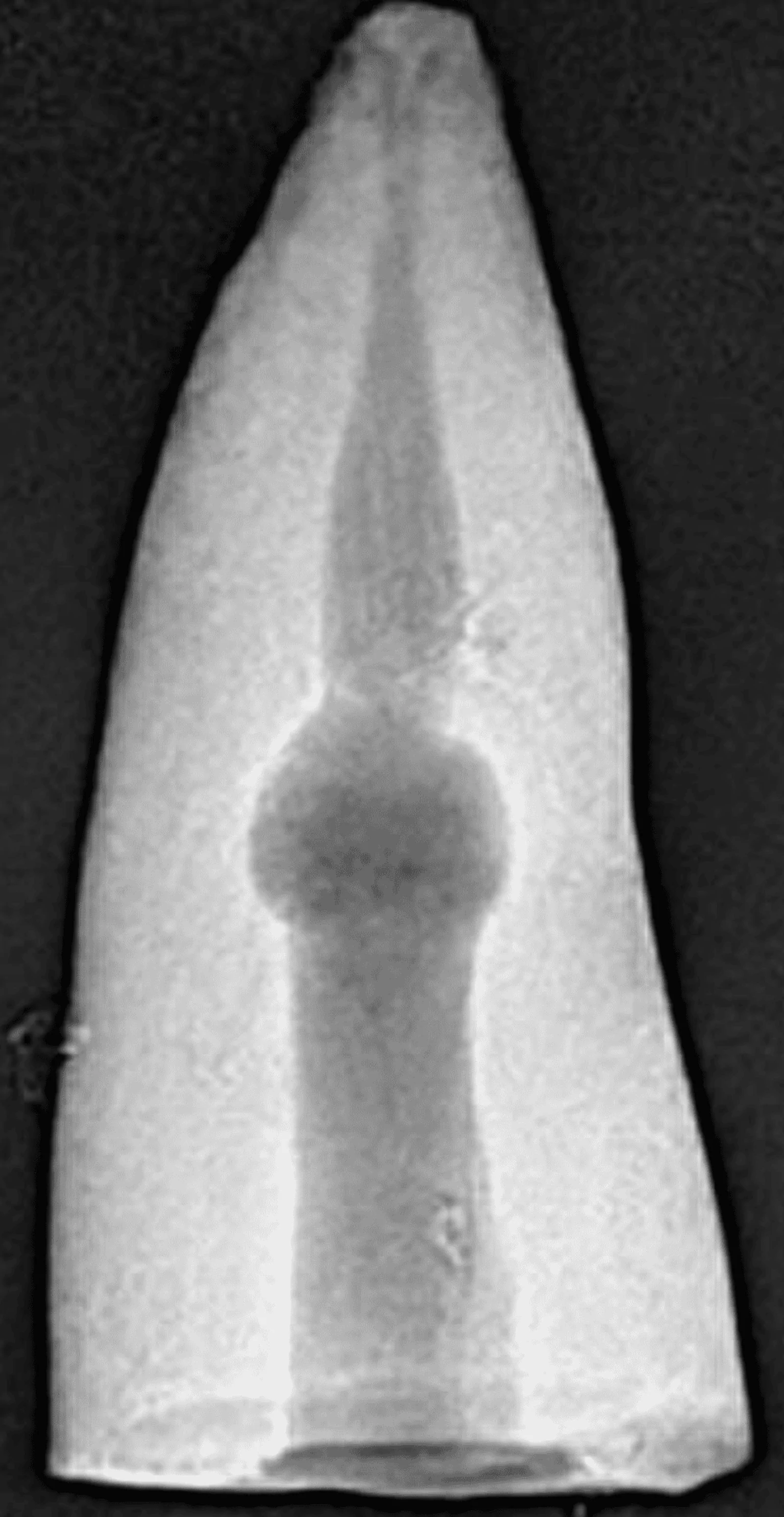
Simulated internal resorption cavity

The roots were sectioned horizontally 14 mm from the apex using a diamond disc under continuous water cooling. Standardized hemispherical cavities measuring approximately 2.5 mm in diameter and 1.25 mm in depth were prepared at the center of each section using a round bur no. 8 to simulate internal resorption defects (Figure 1).

Repair of internal resorption defects

**Figure 2:**
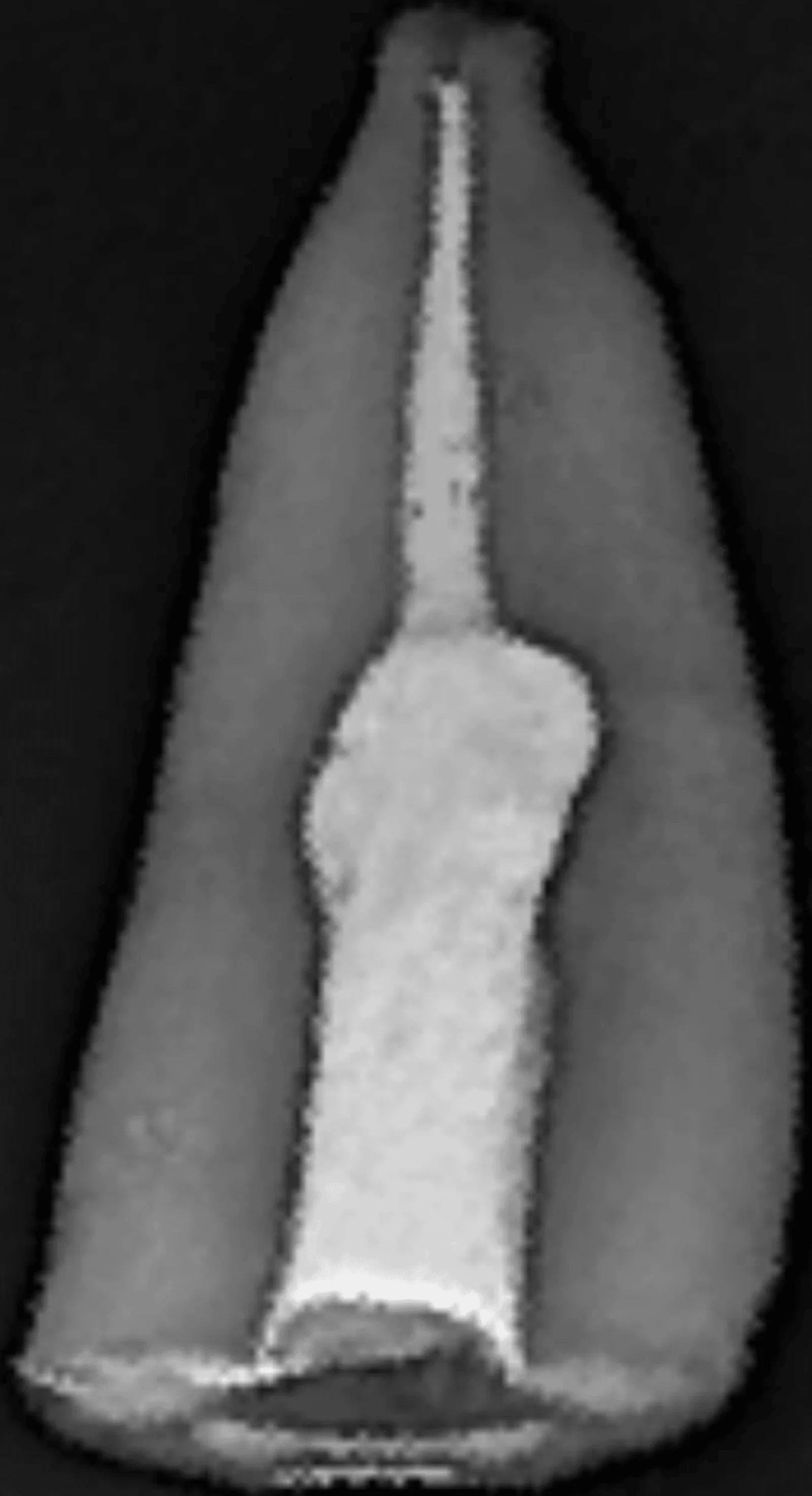
Internal resorption cavity repaired with GP GP: gutta-percha

**Figure 3:**
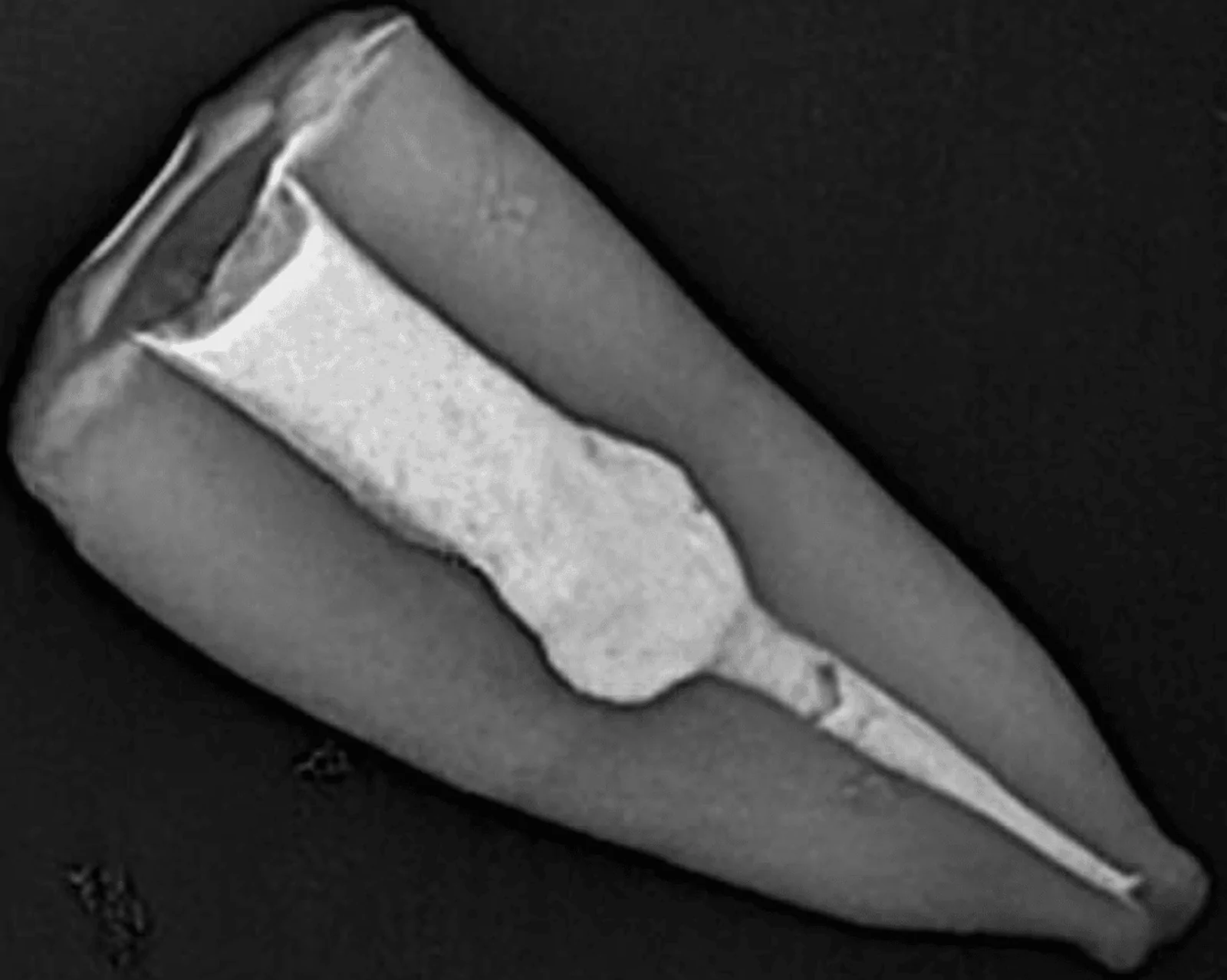
Internal resorption cavity repaired with MTA MTA: mineral trioxide aggregate

**Figure 4:**
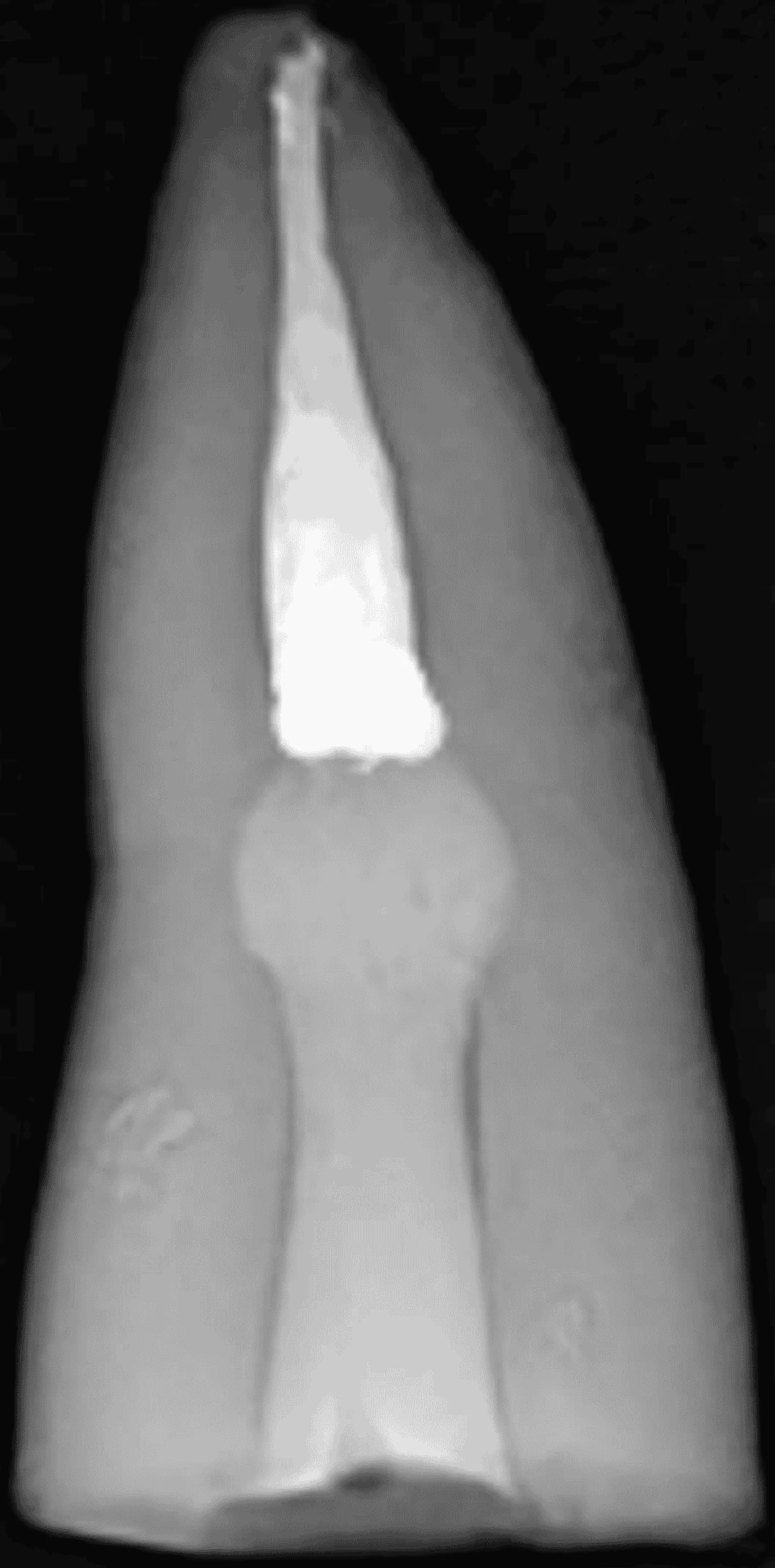
Internal resorption cavity repaired with SFRC SFRC: short fiber-reinforced composite

The canal space apical to the defect was coated with an epoxy resin-based sealer (AH Plus, Dentsply Sirona, Konstanz, Germany) and obturated using thermoplasticized GP (Guilin Woodpecker Medical Instrument Co., Ltd., Guilin, Guangxi, China) to establish a dense, three-dimensional apical seal, following which the specimens were repaired according to the assigned groups. In Group I, the canals and simulated defects were obturated with thermoplasticized GP and an epoxy resin-based sealer (Figure 2). In Group II, the simulated internal resorption defects were repaired with MTA (BioStructure MTA Putty; SafeEndo, New Delhi, India) according to the manufacturer's instructions (Figure 3). In Group III, the simulated defects were restored using SFRC (everX Flow™; GC Dental Products Corp., Tokyo, Japan) (Figure 4). The dentin surfaces were etched for 15 seconds using 35% phosphoric acid (Ultra-Etch™; Ultradent Products, Inc., South Jordan, UT), followed by a 20-second water rinse. Absorbent paper points were used to remove excess moisture. A bonding agent (G-Premio BOND™; GC, Tokyo, Japan) was applied to the canal walls for 10-15 seconds and light-cured. The SFRC was then placed in increments, with each layer light-cured for 10 seconds using an LED unit (>1,200 mW/cm²). After repairing the defect and removing excess material, the two root segments were precisely reassembled with cyanoacrylate adhesive to preserve the original root morphology.

Thermocycling

Following repair of the simulated internal resorption defects, all specimens were subjected to thermocycling to simulate thermal variations encountered in the oral cavity. The specimens underwent thermocycling manually between 5 and 55°C water baths with a dwell time of 30 seconds for 250 cycles.

Mounting of specimens

To simulate the periodontal ligament, the root surfaces were coated with a thin layer of polyvinyl siloxane impression material before embedding vertically in self-cure acrylic resin blocks, leaving approximately 2 mm of the cervical root exposed above the acrylic surface [21,22].

Fracture resistance testing

**Figure 5:**
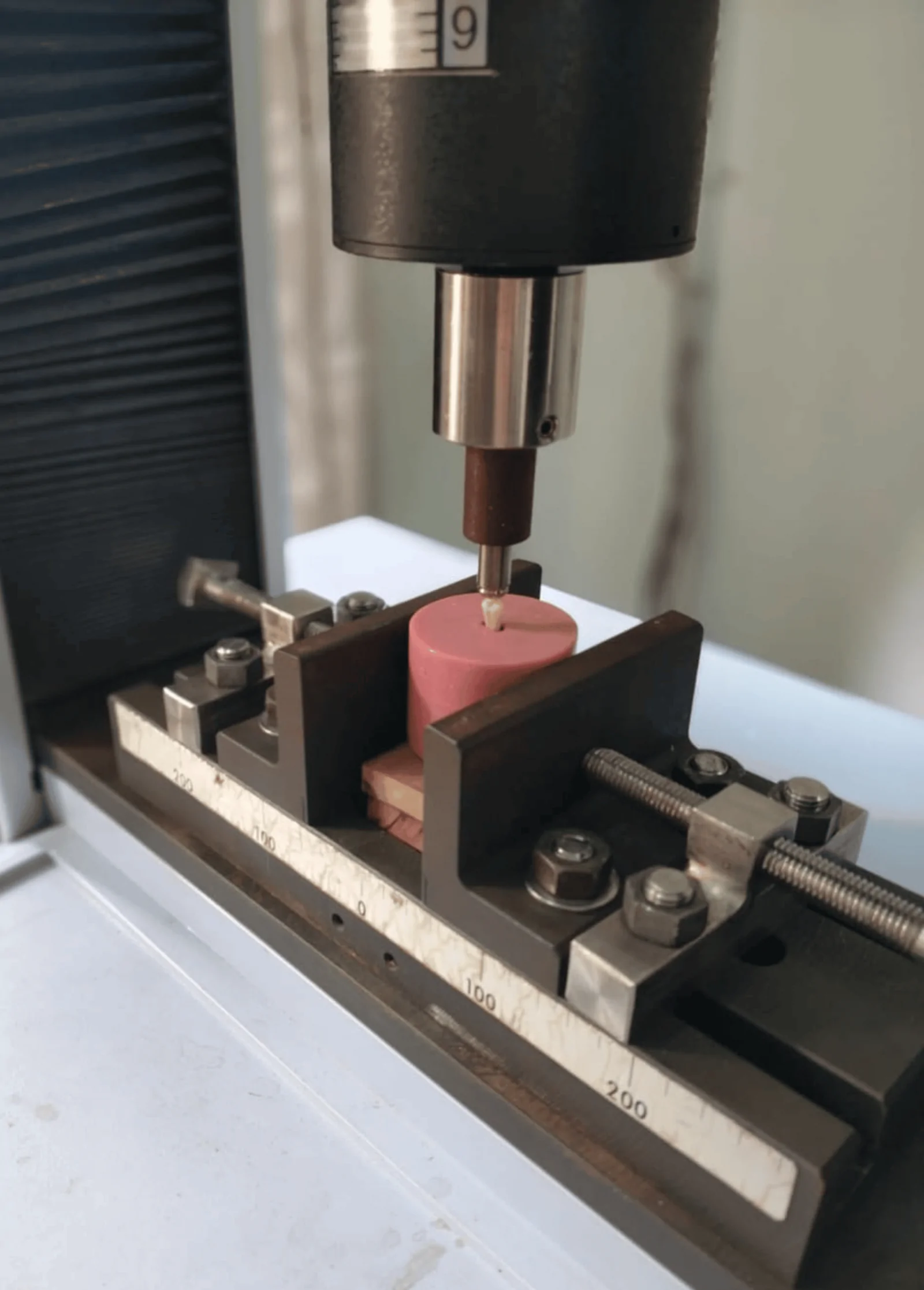
Acrylic blocks containing the embedded teeth mounted in a universal testing machine

All specimens were subjected to fracture testing using a universal testing machine. A compressive load was applied along the long axis of the tooth using a stainless steel spherical tip at a crosshead speed of 1 mm/min until catastrophic fracture occurred. The maximum load required to fracture each specimen was recorded in Newtons (N) (Figure 5).

Statistical analysis

The data were entered into Microsoft Excel (Microsoft® Corp., Redmond, WA) and analyzed using Statistical Product and Service Solutions (SPSS, version 26.0; IBM SPSS Statistics for Windows, Armonk, NY). Descriptive statistics, including mean, standard deviation, minimum, and maximum values, were calculated. Intergroup comparisons were performed using one-way analysis of variance (ANOVA), followed by Tukey's honestly significant difference (HSD) post hoc test for pairwise comparisons. Statistical significance was established at p < 0.05.

## Results

The SFRC group demonstrated the highest mean fracture resistance (1,476.29 ± 100.49 N), followed by the MTA group (1,021.78 ± 65.67 N), whereas the GP (control) group exhibited the lowest mean fracture resistance (829.78 ± 72.74 N) (Table 1). The fracture resistance values ranged from 1,312.45 to 1,612.95 N in the SFRC group; 924.50 to 1,115.45 N in the MTA group; and 721.45 to 945.60 N in the GP group. One-way ANOVA revealed a highly statistically significant difference among the three groups (F = 167.84; p < 0.001), indicating that the root-end filling material significantly influenced the fracture resistance of the teeth. Subsequent Tukey's HSD post hoc analysis demonstrated statistically significant pairwise differences between all groups (p < 0.05) (Table 2). Teeth restored with SFRC exhibited significantly greater fracture resistance than those restored with MTA and GP. Similarly, the MTA group showed significantly higher fracture resistance than the GP control group. These findings suggest that incorporation of SFRC as a root-end filling material considerably enhances the fracture resistance of endodontically treated teeth compared with conventional MTA and GP.

**Table 1:** Comparison of fracture resistance across different groups One-way ANOVA test: *p < 0.05 is statistically significant. GP: gutta-percha; MTA: mineral trioxide aggregate; SFRC: short fiber-reinforced composite

Group	Mean	SD	Min	Max	F-value	p-value
SFRC	1476.29	100.49	1312.45	1612.95	167.84	< 0.001*
MTA	1021.78	65.67	924.5	1115.45		
GP (control)	829.78	72.74	721.45	945.6		

**Table 2:** Tukey's honestly significant difference (HSD) post-hoc multiple comparisons Tukey HSD test: *p < 0.05 is statistically significant. GP: gutta-percha; MTA: mineral trioxide aggregate; SFRC: short fiber-reinforced composite

Group	Comparison	Mean Difference	95% Confidence Interval	p-value
SFRC	MTA	454.52	(364.66, 544.38)	< 0.001*
	GP (control)	646.51	(556.65, 736.37)	< 0.001*
MTA	GP (control)	191.99	(102.13, 281.85)	< 0.001*

## Discussion

Internal root resorption is a progressive pathological process characterized by the loss of intraradicular dentin due to clastic cell activity within an inflamed pulp [1,2]. Although relatively uncommon, untreated internal root resorption may result in extensive destruction of dentinal walls, root perforation, and significant weakening of the tooth structure, thereby increasing its susceptibility to fracture [1]. Successful management of internal root resorption involves not only arresting the resorptive process through effective chemomechanical debridement but also restoring the structural integrity of the weakened tooth using a material capable of providing both an adequate seal and sufficient mechanical reinforcement [7,8]. Therefore, the choice of repair material is critical to the long-term prognosis of teeth affected by internal resorption.

The present study evaluated the fracture resistance of teeth with simulated internal resorption defects repaired using GP, MTA, and SFRC. The results demonstrated statistically significant differences among the three groups (p < 0.001). Teeth repaired with SFRC exhibited the highest fracture resistance, followed by MTA, whereas the GP group demonstrated the lowest fracture resistance.

The superior fracture resistance observed in the SFRC group can be attributed to its unique fiber-reinforced structure. SFRC consists of randomly oriented short E-glass fibers embedded within a resin matrix, which act as crack stoppers by interrupting crack propagation and distributing functional stresses throughout the restoration [14,15]. This mechanism enhances fracture toughness and allows the material to withstand greater occlusal loads before catastrophic failure [15,16]. In addition, the elastic modulus of SFRC is comparable to that of dentin, resulting in more favorable stress distribution within the weakened root and reducing stress concentration at the interface between dentin and the restorative material [17]. These characteristics are particularly advantageous in teeth affected by internal resorption, where substantial dentinal loss compromises root strength [8]. The findings of the present study are consistent with previous investigations demonstrating that fiber-reinforced composites significantly improve the fracture resistance of structurally compromised teeth [18,19,21,23]. Studies by Garoushi et al. and Fráter et al. also reported that the incorporation of short glass fibers effectively arrests crack propagation and enhances the load-bearing capacity of restored teeth [15,19,24].

In this study, MTA demonstrated significantly greater fracture resistance than GP but was inferior to SFRC. MTA has long been regarded as the gold standard for the repair of perforations and internal resorption defects because of its excellent sealing ability, biocompatibility, alkaline pH, bioactivity, and ability to stimulate hard tissue formation [10-12]. These biological properties contribute to favorable healing outcomes and explain its widespread clinical use [12,13]. However, MTA is a calcium silicate-based cement with relatively low flexural strength and fracture toughness. Its brittle nature limits its ability to reinforce roots weakened by extensive dentinal loss, which may account for the lower fracture resistance observed compared with SFRC in the present study [12,25-27].

The GP group exhibited the lowest fracture resistance among all groups. Although GP remains the most widely used obturating material because of its favorable handling properties and clinical success, it does not chemically bond to dentin and contributes minimally to reinforcement of weakened root structure [28,29]. In teeth with internal resorption defects, where dentinal support is already compromised, GP primarily serves as a filling material rather than a reinforcing material [8,29]. Consequently, these specimens fractured under significantly lower loads than those repaired with MTA or SFRC.

An important strength of the present study was the use of standardized simulated internal resorption defects, which enabled reproducible comparison among repair materials. Furthermore, thermocycling was performed before fracture testing to simulate thermal stresses encountered in the oral environment, thereby enhancing the clinical relevance of the findings [30]. While complete root sectioning and cyanoacrylate gluing introduce an artificial interface that disrupts natural dentin continuity, this limitation is offset by its widespread use in endodontic literature to achieve precise defect standardization, and because it was applied uniformly across all groups, it acts as a controlled constant that preserves the validity of the study's comparative results.

From a clinical perspective, preservation of structurally weakened teeth affected by internal root resorption remains a significant challenge. While MTA continues to be the material of choice for cases involving perforating defects because of its superior biological properties, the results of the present study suggest that SFRC may provide greater reinforcement in non-perforating internal resorption defects where restoration of mechanical strength is a primary concern [12,18]. Therefore, material selection should be based not only on biological compatibility but also on the extent of dentinal destruction, location of the defect, and functional demands of the tooth. Within the limitations of this in vitro study, SFRC demonstrated superior reinforcing ability compared with MTA and GP. Further long-term clinical studies and fatigue-loading investigations are required to validate these findings and determine the long-term performance of SFRC in the management of internal root resorption [23].

Limitations

This in vitro study may not fully replicate the clinical conditions of the oral environment. Standardized bur-prepared internal resorption cavities may not accurately represent the morphology of naturally occurring lesions. Furthermore, only mandibular premolars were evaluated; therefore, the findings may not be directly applicable to teeth with different anatomical characteristics. Finally, although static loading provides a simple, standardized way to compare peak strength under controlled conditions, its lack of multi-directional chewing fatigue remains a key limitation that prevents full replication of clinical forces.

## Conclusions

Within the limitations of this in vitro study, SFRC demonstrated significantly higher fracture resistance than MTA and GP. SFRC appears to be the most promising material for reinforcing teeth with internal resorption defects, while MTA provides moderate reinforcement and GP offers the least structural support. Further long-term in vivo studies are required to validate these findings under clinical conditions.
